# Efficacy of non-enhanced computer tomography-based radiomics for predicting hematoma expansion: A meta-analysis

**DOI:** 10.3389/fonc.2022.973104

**Published:** 2023-01-10

**Authors:** Yan-Wei Jiang, Xiong-Jei Xu, Rui Wang, Chun-Mei Chen

**Affiliations:** Department of Neurosurgery, Fujian Medical University Union Hospital, Fuzhou, Fujian, China

**Keywords:** non-enhanced computer tomography, radiomics, hematoma expansion, meta-analysis, spontaneous intracerebral hemorrhage

## Abstract

**Background:**

This meta-analysis aimed to assess the efficacy of radiomics using non-enhanced computed tomography (NCCT) for predicting hematoma expansion in patients with spontaneous intracerebral hemorrhage.

**Methods:**

Throughout the inception of the project to April 11, 2022, a comprehensive search was conducted on PubMed, Embase, and Cochrane Central Register of Controlled Trials. The methodological quality of studies in this analysis was assessed by the radiomics quality scoring system (RQS). A meta-analysis of radiomic studies based on NCCT for predicting hematoma expansion in patients with intracerebral hemorrhage was performed. The efficacy of the radiomics approach and non-contrast CT markers was compared using network meta-analysis (NMA).

**Results:**

Ten articles comprising a total of 1525 patients were quantitatively analyzed for hematoma expansion after cerebral hemorrhage using radiomics. Based on the included studies, the mean RQS was 14.4. The AUC value (95% confidence interval) of the radiomics model was 0.80 (0.76-0.83). Five articles comprising 846 patients were included in the NMA. The results synthesized according to Bayesian NMA revealed that the predictive ability of the radiomics model outperformed most of the NCCT biomarkers.

**Conclusions:**

The NCCT-based radiomics approach has the potential to predict hematoma expansion. Compared to NCCT biomarkers, we recommend a radiomics approach. Standardization of the radiomics approach is required for further clinical implementation.

**Systematic review registration:**

https://www.crd.york.ac.uk/PROSPERO/display_record.php?RecordID=324034, identifier [CRD42022324034].

## 1 Introduction

Intracerebral hemorrhage is a life-threatening and costly disorder that accounts for 10–15% of all strokes (1). Hematoma expansion is an independent risk factor for poor neurological outcomes. Predictions of hematoma expansion risks can help to stratify patients. Previous studies have reported that spot signs are a good predictor of hematoma expansion (2, 3). Nevertheless, the application of spot signs is limited because computed tomography angiography (CTA) and contrast-enhanced CT are not routinely performed in the emergency department. Non-enhanced CT (NCCT) is most commonly used for intracerebral hemorrhage imaging. Several studies have reported that radiological markers extracted from NCCT, including the black hole, satellite, and blend signs, are related to hematoma expansion (2). However, the extraction of radiomic markers is time-consuming and heterogeneous. Further, the accuracy of radiomic markers may depend on the experience of the clinician who reads the medical images.

**Figure 1:**
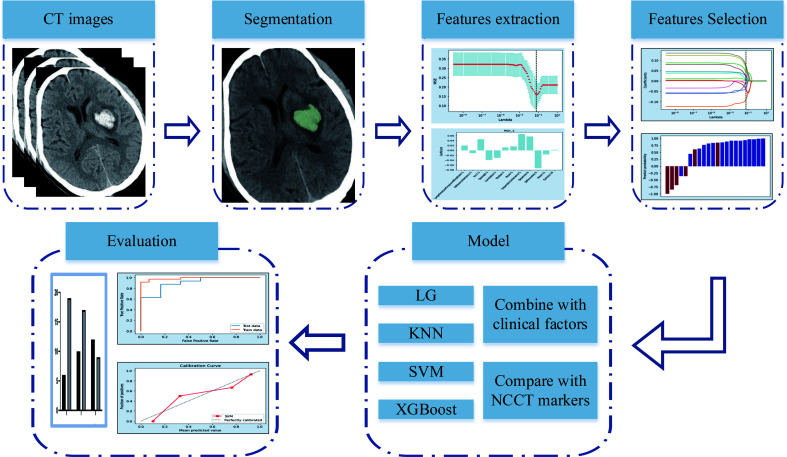
Flowchart of (NCCT-based radiomics. NCCT: non-enhanced computed tomography.

Radiomics is a new method for the quantitative analysis of medical images (4). Radiomics analysis was initially implemented in the mining of medical images related to oncology. Recently, the radiomics approach has been applied in non-oncological fields (5). An increasing number of studies have used an NCCT-based radiomics approach to predict hemorrhage expansion(**Figure 1**) (4, 6, 7). However, data on the predictive efficacy of radiomics methods remain insufficient for further implementation.

This meta-analysis aimed to determine whether NCCT-based radiomics approaches are effective for predicting hematoma expansion. Radiomics quality scoring (RQS) was used to determine the quality of the studies included in the meta-analysis (8). Network meta-analysis (NMA) was employed to synthesize diagnostic test accuracy data in order to assess the efficacy of different diagnostic tests (9, 10). We compared the efficacy of common NCCT markers and radiomics approaches for predicting intracerebral hemorrhage expansion using NMA.

## 2 Methods

### 2.1 Literature search and study selection

This study was conducted according to the Preferred Reporting Items for Systematic Reviews and Meta-Analysis (PRISMA) statement (**eTable 1**) (11). This study was registered with PROSPERO (CRD42022324034). PubMed, Embase, and the Cochrane Central Register of Controlled Trials were searched thoroughly from inception to April 11, 2022 (**eTable 2**) for articles in English. References to relevant published articles were also searched to obtain the desired articles.

After pooling the search results from the three databases and removing duplicate articles, the abstracts and titles of the articles were screened independently by two researchers. Eligible articles were identified by a comprehensive reading of the full text. We included all eligible radiomics articles that used non-enhanced CT to assess hematoma expansion in patients with intra-cerebral hemorrhage. Articles that met one or more of the following criteria were excluded: (1) conference abstracts, reviews, letters, case reports, and case series studies with sample sizes < 10; (2) in multiple studies using the same population, only the study with the largest dataset was included; (3) non-human studies; (4) secondary intra-cerebral hemorrhage; (5) intraventricular hemorrhage; and (6) studies without comparison. All inconsistencies were resolved by negotiation or by a third investigator.

### 2.2 Data extraction

Eligible articles contained information that was independently extracted by two researchers, including authors, year of publication, sample size, number of cases in the training and validation sets, study population, study design, study country, number of institutions, composition of model construction, mode of visualization, interval image examination, research topic, segmentation software, method of extraction of imaging histology features, validation method, method of screening variables, final study characteristics, sensitivity (Se), specificity (Sp), true positives (TPs), false positives (FPs), true negatives (TNs), false negatives (FNs), diagnostic accuracy rate (DAR), diagnostic odds ratio (DOR), number of hematoma expansions, and non-expansions. All inconsistencies were resolved by negotiation or by a third investigator.

### 2.3 Quality assessment

All eligible studies were assessed for bias using the QUADAS-2 tool for diagnostic meta-analyses (12). Four key domains were assessed, including flow and timing, reference standards, index tests, and patient selection. Three main domains were assessed using the Applicability Concerns Test. Risk of bias was categorized as low, high, or unclear. When all domains were rated as yes, the risk was considered low. A potential risk of bias existed when any of the domains was rated no. The unclear classification only applied when there were insufficient data to report. The 16 components of RQS were used to assessed the quality of radiomics studies (8). Reviewers scored each component and summed up the scores. The procedures for scoring each component have been described previously.

### 2.4 Outcome measures

We performed a synthetic analysis of TP, FP, TN, and FN indicators of eligible articles using a diagnostic meta-analysis. Comparative analyses were performed for Se, Sp, positive predictive value (PPV), negative predictive value (NPV), DAR, and DOR. Articles that did not provide the four indicators TP, FP, TN, and FN were calculated using the number of cases of hematoma expansion and non-expansion, combined with Se and Sp, using Review Manager 5.4.1. (**eTable 3**).

Se refers to the proportion of positive cases detected within the group diagnosed with disease by the gold standard; a higher Se indicates a lower chance of a missed diagnosis. Sp refers to the proportion of negative tests within the group diagnosed as disease-free by the gold standard; a higher Sp indicates a lower chance of misdiagnosis. PPV reflects the proportion of individuals with a positive screening test result who are actually sick. NPV reflects the proportion of individuals with a negative screening test result who do not actually have the disease. DAR is defined as the proportion of all cases detected as TPs and TNs by clinical diagnostics within all cases. A higher DOR value indicates that the diagnostic test is more effective at distinguishing between patients and non-patients.

## 3 Data Synthesis

### 3.1 Diagnostic meta-analysis to evaluate diagnostic test accuracy

Diagnostic test accuracy indicators, such as Se, Sp, PPV, NPV, DAR, and DOR, were synthesized using a meta-analysis based on a random-effects model. Forest plots were used to represent the effect values (odds ratio, OR) and 95% confidence intervals (CIs). Evaluation of the screening biomarkers (radiological features or radiomics) was based on summary receiver operating characteristic (sROC) curves and areas under the curve (AUCs), whereby a larger AUC indicates better model performance. The Cochrane Q test and I^2^ were used to measure the heterogeneity of the outcomes. The robustness of the results was evaluated, and sources of heterogeneity were explored by omitting each included article one by one in the pooled analysis. Publication bias was evaluated using funnel plots. A p-value < 0.05 for the Q test or I^2^ > 50% indicated the possibility of significant heterogeneity.

### 3.2 NMA

Studies that included a comparison of radiomics and radiological markers were used for the NMA. We used NMA to evaluate the diagnostic value of all radiological features and radiomics evaluating hematoma expansion in cerebral hemorrhage in all eligible studies to estimate the OR and 95% CI for predicting hematoma expansion for Se, Sp, PPV, and NPV in eligible articles, and to summarize the rank order for all screening biomarkers.

The implementation of the NMA was based on a Bayesian model using Markov chain Monte Carlo simulation methods (MCMC), where the calculated prior distribution and likelihood values were substituted into MCMC, and the parameters were adjusted to three chains and 5000 burn-ins using a random-effects model with 50,000 iterations and an interval of 5. An optimal fit state of the convergent posterior distribution was obtained, minimizing the variation of the MCMC error and deviation information criterion to stabilize the ending (13, 14). Trace plots and density distribution plots were used to assess aggregation. We constructed network plots for each outcome measure separately. The plot points represented different screening biomarkers, point sizes indicated the total sample size for each feature, and line thickness represented the number of studies that were conducted between the two points connected. The OR values and 95% CIs between different predictors were represented using forest plots. To better compare diagnostic efficacy, surface under the cumulative ranking (SUCRA) was used to calculate the rankings of the predictors (15). SUCRA values ranged between 0 and 1, with larger values representing higher rankings and diagnostic efficacies.

In hypothesis testing for the NMA, we determined homogeneity and transferability by assessing the baseline characteristics of the included articles, methodological and statistical approaches, and agreement of the same predictor across articles. For consistency tests, we explored local heterogeneity using nodal splits and the deviance information criterion for consistency and inconsistency models (DIC) (16). A smaller difference between DIC values of the two models indicated good agreement.

As most of the articles constructed the model using the training set, we only used data from the validation set for synthetic analysis. Model performance was verified using the validation set. Articles that were not categorized into the training and validation sets were analyzed as validation sets.

All data synthesis was conducted using R version 4.1.2 (“meta” package for diagnostic meta-analysis to evaluate diagnostic test accuracy; “gemtc” package for NMA) and Review Manager 5.4.1.

## 4 Results

**Figure 2:**
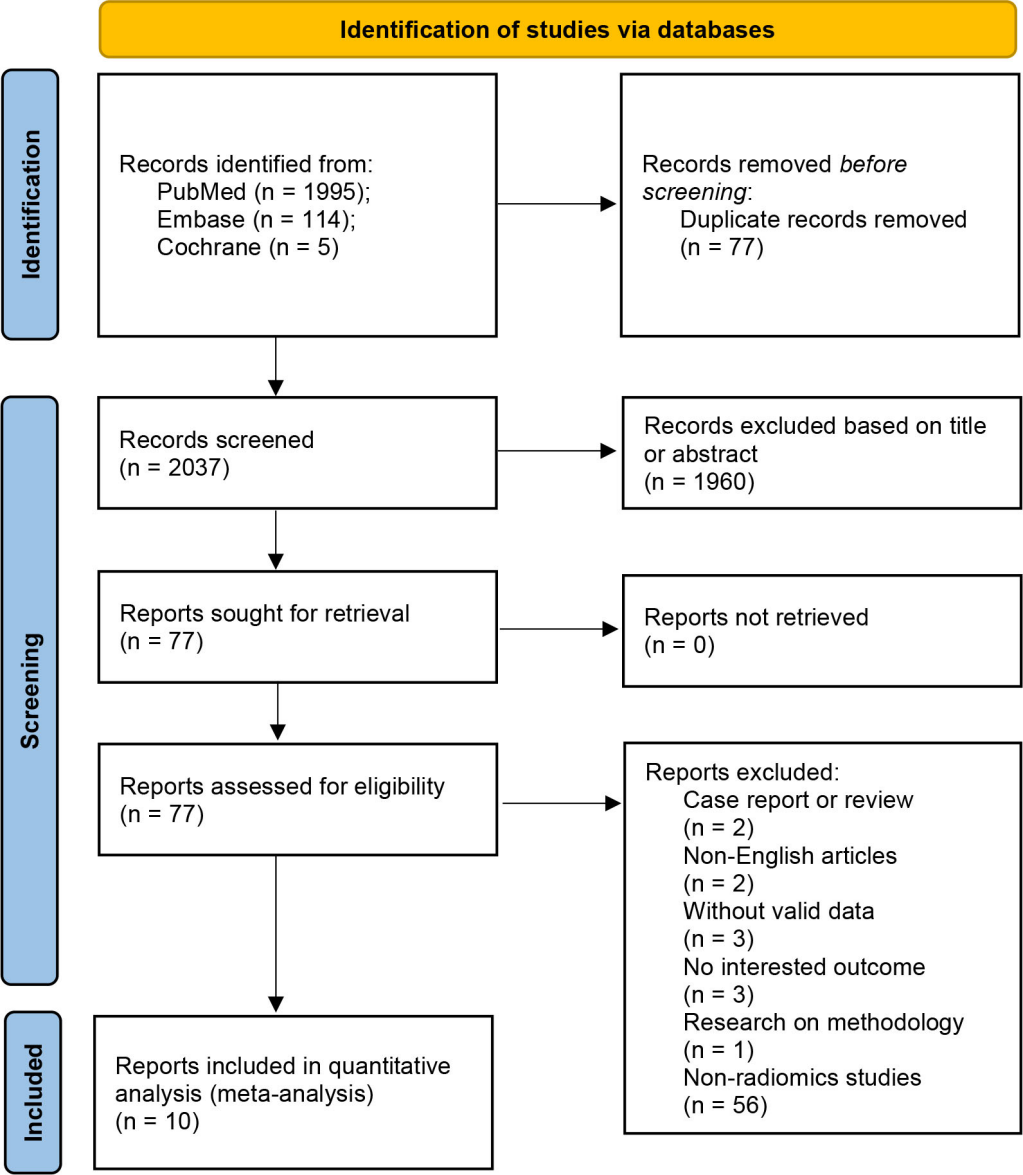
Flowchart for study selection.

A comprehensive search of 2114 articles was conducted, and 2037 articles were screened after excluding duplicates. Of the 77 full-text articles searched, 67 were excluded according to the inclusion and exclusion criteria (**Figure 2**). Ten articles comprising a total of 4929 patients were finally included in the meta-analysis, five of which were used for the NMA. (**Table 1**). The causes of cerebral hemorrhage were spontaneous intracerebral hemorrhage and hypertensive intraparenchymal hematoma. A total of 1391 (28.22%) patients had hematoma expansion. The radiological features or radiomics analysis performed included radiomics model, radiological model, radiomics-radiological model, black hole sign, blend sign, heterogeneity, hypodensity, irregular shape, island sign, midline shift, satellite sign, and swirl sign.

**Table 1:** Baseline characteristics of included studies.

Author	Year	Sample size	HE	non-HE	Study design	Study location	Imaging modality	Research question
Chen et al	2021	1153	236	917	Retrospectively	China	NCCT	To compare the predictive performance for HE among clinical model, radiomics model, and hybrid model.
Duan et al	2022	108	54	54	Retrospectively	China	NCCT	To predict HE by using different machine learning methods and to determine the best radiomics model.
Li et al	2019	167	42	125	Retrospectively	China	NCCT	To quantify the heterogeneity of hematomas in order to find more quantitative, sensitive, and accurate indicators for predicting HE.
Ma et al	2019	254	58	196	Retrospectively	China	NCCT	To explore the feasibility of predicting hematoma expansion at acute phase *via* a radiomics approach.
Pszczolko-wski et al	2021	1732	474	1258	Retrospectively	England	NCCT	To investigate the use of NCCT radiomics-based features and generalized linear models for prediction of both HE and poor functional outcome
Song et al	2021	261	110	151	Retrospectively	China	NCCT	To determine whether NCCT) models based on multivariable, radiomics features, and machine learning (ML) algorithms could further improve the discrimination of early hematoma expansion (HE) in patients with spontaneous intracerebral hemorrhage.
Xia et al	2022	376	108	268	Retrospectively	China	NCCT	To identify supratentorial spontaneous intracerebral hemorrhage patients with HE on admission
Xie et al	2020	251	108	143	Retrospectively	China	NCCT	To predict hematoma expansion and to compare the predictive performance with conventional radiological feature-based model
Zhan et al	2021	313	44	269	Retrospectively	China	NCCT	To predict HE and the short- term outcomes in patients with small hematomas.
Zhu et al	2021	314	157	157	Retrospectively	China	NCCT	To evaluate HE prediction in the perihematomal region using radiomics technology and compare its predictive performance with the intra-hematomal radiomics signature.

HE: hematoma expansion; NCCT: non-enhanced computed tomography.

Based on the QUADAS-2 tool for assessing bias and applicability (**eFigure 1**), the overall quality of the included articles was satisfactory. For some studies, we could not determine whether the included patients were consecutive or randomized (n=8) and whether a blinded approach and diagnostic thresholds were used for analysis (n=3); these articles were therefore categorized as unclear.

Based on RQSs (**Table 2**), the included articles were generally of low quality. In ten studies, the mean score was 40% (range, 30.1–69.4%), and one study scored above 50%. The protocols for image acquisition reported in most studies were well-documented. Most studies (70%) used manual segmentation (which is usually performed by an expert drawing ROIs), and three (30%) used semi-automatic segmentation (which combines manual segmentation with some algorithms). Two (20%) of the studies integrated clinical data into radiomic models and suggested that this could improve prediction accuracy.

**Table 2:** Radiomics quality scores.

Author	①	②	③	④	⑤	⑥	⑦	⑧	⑨	⑩	⑪	⑫	⑬	⑭	⑮	⑯	Total score	Mean score (%)
Song	1	1	0	0	3	1	0	0	1	1	0	2	2	2	0	0	14	38.9
Li	1	1	0	0	3	0	0	0	0	0	0	2	2	2	0	0	11	30.6
Ma	1	1	0	0	3	0	0	0	0	0	0	2	2	2	0	0	11	30.6
Xie	1	1	0	0	3	1	1	0	0	0	0	2	2	2	0	0	13	36.1
Chen	1	1	0	0	3	1	1	0	1	1	0	2	2	2	0	0	15	41.7
Pszczolkowski	0	0	0	0	3	1	1	1	1	1	7	4	2	2	0	2	25	69.4
Zhan	1	0	0	0	3	1	1	0	1	0	0	2	2	2	0	0	13	36.1
Zhu	0	1	0	0	3	1	1	0	1	1	0	2	2	2	0	0	14	38.9
Duan	1	1	0	0	3	1	0	0	1	0	0	2	2	2	0	0	13	36.1
Xia	1	1	0	0	3	1	0	0	1	1	0	3	2	2	0	0	15	41.7

①:Image protocol quality; ②: Multiple segmentations; ③: Phantom study on all scanners; ④: Imaging at multiple time points; ⑤: Feature reduction or adjustment for multiple testing; ⑥: Multivariable analysis with non-radiomics features; ⑦: Detect and discuss biological correlates; ⑧: Cutoff analyses; ⑨: Discrimination statistics; ⑩: Calibration statistics; ⑪: Prospective study registered in a trial database; ⑫: Validation; ⑬: Comparison to gold standard; ⑭: Potential clinical utility; ⑮: Cost effectiveness analysis; ⑯: Open science and data.

### 4.1 Diagnostic test meta-analysis

#### 4.1.1 Radiomics model

**Figure 3:**
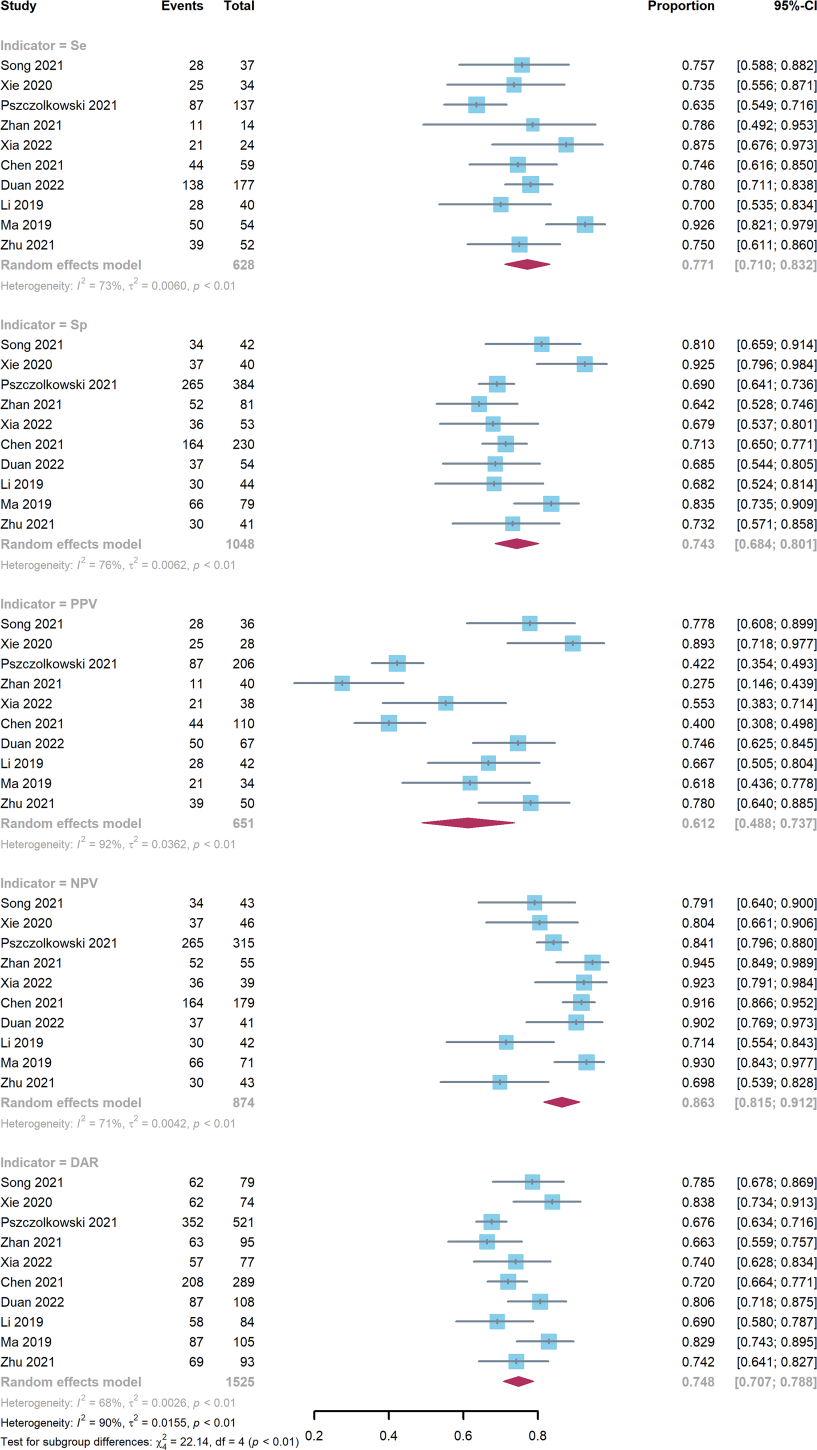
Forest plot of radiomics model. CIs: confidence intervals; DAR: diagnostic accuracy rate; NPV: negative predictive value; PPV: positive predictive value; Se: sensitivity; Sp: specificity.

Ten studies comprising a total of 1525 patients were quantitatively analyzed for hematoma expansion after cerebral hemorrhage using the radiomics method (4, 6, 7, 17–23). The pooled Se, Sp, PPV, NPV, and DAR were 0.771 (0.710-0.832), 0.743 (0.684-0.801), 0.612 (0.448-0.737), 0.863 (0.815-0.912), and 0.748 (0.707-0.788), respectively (**Figure 3**). The synthetic DOR was 9.85 (6.01-16.12) (**eFigure 2**).

### 4.2 sROCs and AUCs

The sROC curves demonstrated similar model performance for the radiomics and radiomics-radiological models, and better performance than that of the other screening biomarkers (**eFigure 3**). The AUC value (95% CI) of the radiomics model was 0.80 (0.76-0.83) (**eFigure 4**). However, other biomarkers were not available due to the limited number of studies that obtained AUC values and 95% CIs.

### 4.3 Sensitivity analysis and publication bias

For most diagnostic indictors, the Cochrane’s Q (p<0.05) and I^2^ (I^2^>50%) tests revealed significant heterogeneity. However, no significant changes were observed in the Se, Sp, PPV, NPV, and DAR values after article-by-article exclusion, suggesting the robustness of the outcomes and relatively low potential heterogeneity (**eFigures 5**). Funnel plots for different diagnostic indicators of the radiomics model suggested publication bias (**eFigure 6**).

### 4.4 NMA

**Figure 4:**
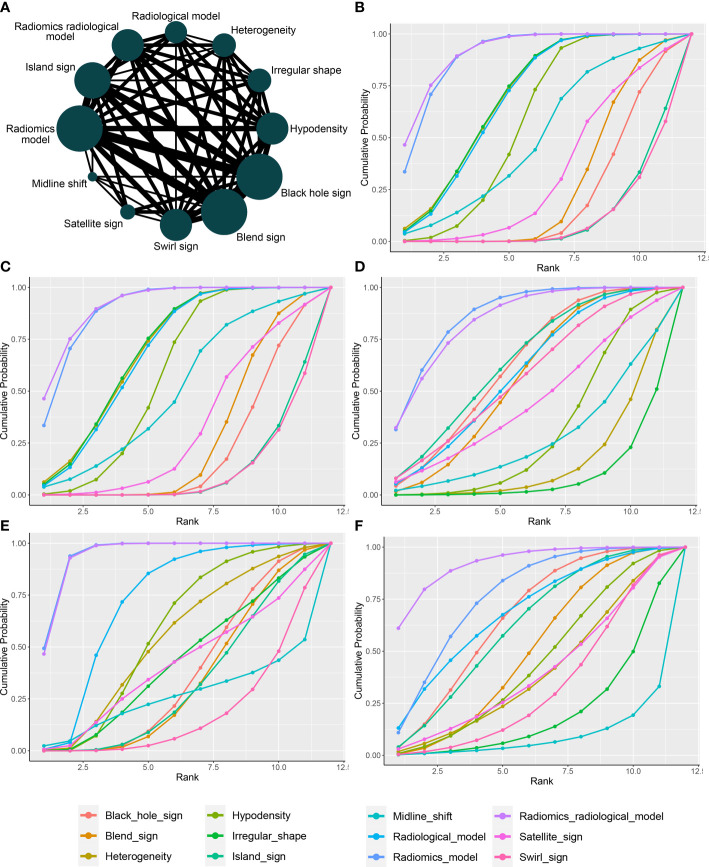
Network plot and SUCRA of biomarkers for ranking the accuracy of diagnosis. **(A)** The network plot of Bayesian network meta-analysis; **(B)** sensitivity; **(C)** specificity; **(D)** positive predictive value; **(E)** negative predictive value; **(F)** diagnosis accuracy rate. SUCRA: Surface under the cumulative ranking curve.

**Figure 5:**
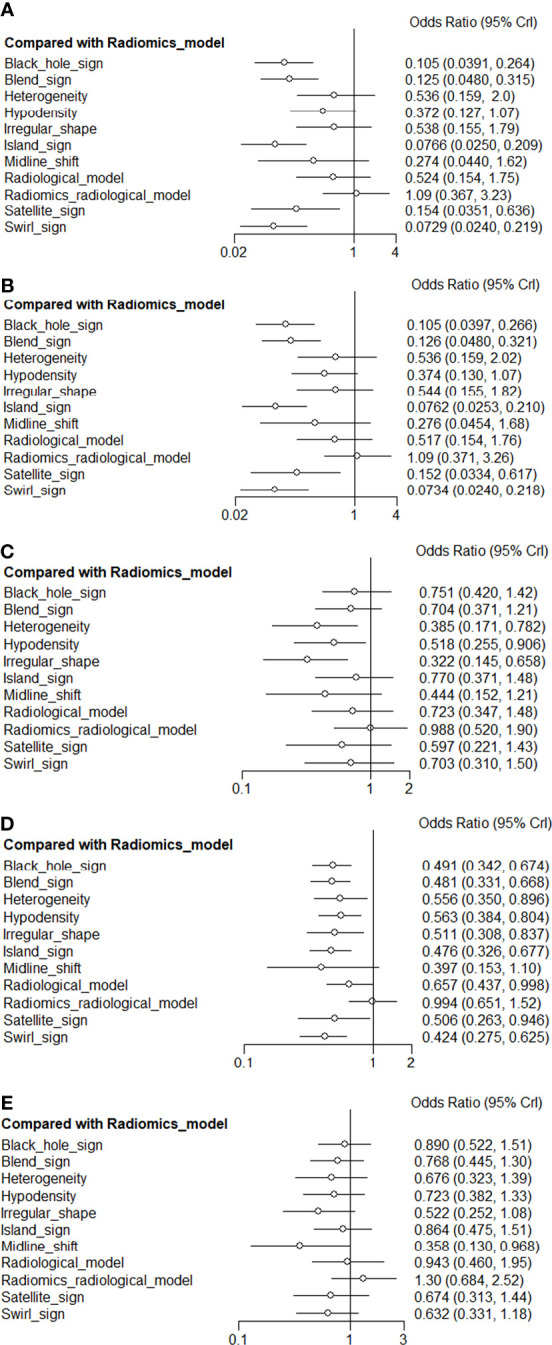
Network forest plot for biomarkers compared with radiomics model. Crl: credible interval.

**Figure 4A** presents a network plot of the indicators involved in the composition. In the NMA, 846 patients from five articles were included (4, 6, 7, 21, 22). The results synthesized according to Bayesian NMA revealed that the predictive ability of the radiomics model outperformed most of the NCCT biomarkers (**Figure 5**). According to SUCRA (**eTable 4**), both radiomics and radiomics-radiological models were ranked in the top two for Se, Sp, PPV, NPV, and DAR. SUCRA curves are presented in **Figures 4B–F**.

The results of the node-splitting method revealed good consistency (p > 0.05) in most of the direct or indirect comparisons (**eFigure 7**). The difference between the DIC values of both the consistent and inconsistent models did not exceed 5 and exhibited good consistency (**eTable 5**).

## 5 Discussion

This meta-analysis examined the utility of NCCT-based radiomics methods to predict hematoma expansion. Our analysis indicated that the radiomics approach demonstrated potential for the prediction of hematoma expansion. Despite these promising results, the relatively low RQSs of the included studies revealed that the radiomics approach was suboptimal for clinical application. Additionally, our analysis revealed that the aggregated Se, Sp, and AUC of the radiomics model outperformed those of the radiological biomarkers.

**Figure 6:**
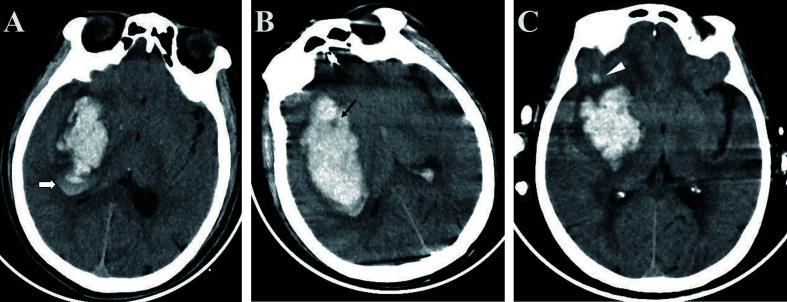
Examples of biomarkers on non-enhanced computed tomography **(A)** The blend sign is defined as an area with relatively low attenuation adjacent to an area with high attenuation (white arrow); **(B)** The black hole sign represents a low-density area within a hematoma with high density (black arrow); **(C)** The satellite sign represents a small lesion completely separate from the main hemorrhage visible in at least one slice (white triangle).

The results of our meta-analysis demonstrated that NCCT-based radiomics is a feasible approach for stratifying the risk of spontaneous intracerebral hemorrhage (21, 24–27). Hematoma expansion is associated with clinical outcomes of spontaneous intracerebral hemorrhage. Though there is currently no definitive therapeutic strategy for prevention of hematoma expansion, we believe that the HE is an appealing target for medical intervention, as it may ultimately help some patients with intracerebral hemorrhages. The CTA spot sign is useful for stratifying patient risk and providing appropriate treatment (3, 28). However, in most medical centers in China, immediate CTA is not routinely performed, thus limiting the implementation of spot signs. NCCT, which is cheaper and more convenient, is the most commonly used method for diagnosing intracerebral hemorrhage. Previous studies have reported that NCCT biomarkers, including the blend sign, black hole sign, and satellite sign, can be used to predict the risk of hematoma expansion. According to Li et al. (29), the blend sign (**Figure 6A**), which is defined as an area that has relatively low attenuation adjacent to an area with high attenuation, showed 39.3% sensitivity and 95.5% specificity for predicting hematoma growth. The black hole sign (**Figure 6B**), which represents a low-density area within a hematoma with high density, is reported to have sensitivity of 31.9% and specificity of 94.1% (30). One of the attributes of the satellite sign (**Figure 6C**) is shape irregularity. A comparative study by Shakya (31) showed that the areas under the curve for the black hole sign and the satellite sign were 63.4% and 67%, respectively. The relatively low efficacy of the predictive ability of a single NCCT biomarker restricts their clinical utility. In contrast, the radiomics studies included in our meta-analysis exhibited superior performance.

Compared to radiological markers, the radiomics method, which is based on mathematical calculation, is a more stable method to predict the risk of hematoma expansion (4, 32). The definition of radiological markers can be inconsistent, which has hindered clinical implementation of NCCT biomarkers. In this regard, a degree of overlap exists between NCCT markers (33). Moreover, the identification of radiological signs depends on the reader’s experience. Our meta-analysis demonstrated that the efficacy of NCCT markers was suboptimal for implementation in clinical practice. Notably, radiomics features may quantitatively reflect the corresponding NCCT biomarkers. Although a limited number of studies was included, our results demonstrated that the radiomics method outperformed radiological biomarkers for predicting hematoma expansion.

Despite its potential, the radiomics method is relatively novel, and non-standardized imaging protocols remain commonplace. RQS was designed to measure the quality of radiomics research (8, 34). The RQSs, which includes 16 items, can be used to assess the quality of radiomics studies. Although evolving rapidly, research applying radiomics must comply with certain basic principles. For instance, data obtained from other institutions is considered to be more independent and therefore more reliable when compared to data obtained internally. External validation of models is crucial for ensuring their generalizability. Indeed, the lack of external validation is the main factor for a low RQS (35). Standardization of high-quality image-extracted data may be helpful for clinical decision support systems (36, 37).

This study had several limitations. Meta-analysis had the limitation of heterogeneity among studies included. Based on the methods used for image reconstruction, feature extraction, and algorithms used, there were considerable differences between the included studies. Second, there was a limited number of eligible studies in the meta-analysis, possible because the relative improvement in performance of the radiomics method was overestimated, and the radiological markers were understated. Third, radiomics studies are generally of low quality, most lack external validation, and promising results from radiomics should be interpreted with caution. Higher-level evidence from clinical trials is necessary for clinical implementation of radiomics approaches.

In conclusion, our meta-analysis highlights the potential of NCCT-based radiomics approaches to predict hematoma expansion. In this regard, we recommend a radiomics approach over NCCT markers. Nevertheless, standardization of radiomics approaches is necessary for further clinical application, and further multicenter prospective studies with stricter designs are warranted to verify our findings.

## Data availability statement

The original contributions presented in the study are included in the article/**Supplementary Material**. Further inquiries can be directed to the corresponding author.

## Author contributions

Y-WJ and X-JX contributed to the study conception and design. Material preparation, data collection and analysis were performed by Y-WJ, X-JX, and C-MC. The first draft of the manuscript was written by Y-WJ and all authors commented on previous versions of the manuscript. All authors read and approved the final manuscript.
