# Supplementary material for: Efficacy of non-enhanced computer tomography-based radiomics for predicting hematoma expansion: A meta-analysis
Source: Front Oncol. 2023 Jan 10;12:973104. doi: 10.3389/fonc.2022.973104 (PMC9872032; doi:10.3389/fonc.2022.973104)
Supplement: Supplementary file 1 [file DataSheet_1.docx]

Supplementary materials

Content

[eTable 1. Checklist of the PRISMA extension for network meta-analysis. 2](#_Toc110670256)

[eTable 2. Search strategy 6](#_Toc110670257)

[eTable 3. The formula of the outcome measures. 8](#_Toc110670258)

[eTable 4. The ranking result and the value of surface under the cumulative ranking curve (SUCRA). 9](#_Toc110670259)

[eTable 5. Comparisons of the fit of consistency and inconsistency models. 10](#_Toc110670260)

[eFigure 1. Risk of bias assessment for included studies (QUADAS-2 tool). 11](#_Toc110670261)

[eFigure 2. The forest plot of diagnostic odd ratio for diagnostic test meta-analysis. 12](#_Toc110670262)

[eFigure 3. Summary receiver operating characteristic curves (sROC) of the screening indicators. 13](#_Toc110670263)

[eFigure 4. Summary receiver operating characteristic curves (sROC) fo radiomics model. 14](#_Toc110670264)

[eFigure 5. Sensitivity analysis for radiomics by omitting each included article one by one. 15](#_Toc110670265)

[eFigure 6. Funnel plot of publishing bias for radiomics model. 17](#_Toc110670266)

[eFigure 7. Node-split for assessing local heterogeneity. 20](#_Toc110670267)

# eTable 1. Checklist of the PRISMA extension for network meta-analysis.

| **Section and Topic** | **Item #** | **Checklist item** | **Location where item is reported** |
| --- | --- | --- | --- |
| **TITLE** | | |  |
| Title | 1 | Identify the report as a systematic review. | 1 |
| **ABSTRACT** | | |  |
| Abstract | 2 | See the PRISMA 2020 for Abstracts checklist. | 1-2 |
| **INTRODUCTION** | | |  |
| Rationale | 3 | Describe the rationale for the review in the context of existing knowledge. | 2 |
| Objectives | 4 | Provide an explicit statement of the objective(s) or question(s) the review addresses. | 2 |
| **METHODS** | | |  |
| Eligibility criteria | 5 | Specify the inclusion and exclusion criteria for the review and how studies were grouped for the syntheses. | 2-3 |
| Information sources | 6 | Specify all databases, registers, websites, organisations, reference lists and other sources searched or consulted to identify studies. Specify the date when each source was last searched or consulted. | 2 |
| Search strategy | 7 | Present the full search strategies for all databases, registers and websites, including any filters and limits used. | 2-3,  eTable 2 |
| Selection process | 8 | Specify the methods used to decide whether a study met the inclusion criteria of the review, including how many reviewers screened each record and each report retrieved, whether they worked independently, and if applicable, details of automation tools used in the process. | 2-3 |
| Data collection process | 9 | Specify the methods used to collect data from reports, including how many reviewers collected data from each report, whether they worked independently, any processes for obtaining or confirming data from study investigators, and if applicable, details of automation tools used in the process. | 3 |
| Data items | 10a | List and define all outcomes for which data were sought. Specify whether all results that were compatible with each outcome domain in each study were sought (e.g. for all measures, time points, analyses), and if not, the methods used to decide which results to collect. | 3-4 |
|  | 10b | List and define all other variables for which data were sought (e.g. participant and intervention characteristics, funding sources). Describe any assumptions made about any missing or unclear information. | 3-4 |
| Study risk of bias assessment | 11 | Specify the methods used to assess risk of bias in the included studies, including details of the tool(s) used, how many reviewers assessed each study and whether they worked independently, and if applicable, details of automation tools used in the process. | 3 |
| Effect measures | 12 | Specify for each outcome the effect measure(s) (e.g. risk ratio, mean difference) used in the synthesis or presentation of results. | 3-4 |
| Synthesis methods | 13a | Describe the processes used to decide which studies were eligible for each synthesis (e.g. tabulating the study intervention characteristics and comparing against the planned groups for each synthesis (item #5)). | 4 |
|  | 13b | Describe any methods required to prepare the data for presentation or synthesis, such as handling of missing summary statistics, or data conversions. | 4 |
|  | 13c | Describe any methods used to tabulate or visually display results of individual studies and syntheses. | 4 |
|  | 13d | Describe any methods used to synthesize results and provide a rationale for the choice(s). If meta-analysis was performed, describe the model(s), method(s) to identify the presence and extent of statistical heterogeneity, and software package(s) used. | 4 |
|  | 13e | Describe any methods used to explore possible causes of heterogeneity among study results (e.g. subgroup analysis, meta-regression). | 4 |
|  | 13f | Describe any sensitivity analyses conducted to assess robustness of the synthesized results. | 4 |
| Reporting bias assessment | 14 | Describe any methods used to assess risk of bias due to missing results in a synthesis (arising from reporting biases). | 4 |
| Certainty assessment | 15 | Describe any methods used to assess certainty (or confidence) in the body of evidence for an outcome. | 3-4 |
| **RESULTS** | | |  |
| Study selection | 16a | Describe the results of the search and selection process, from the number of records identified in the search to the number of studies included in the review, ideally using a flow diagram. | 5,  Figure 2 |
|  | 16b | Cite studies that might appear to meet the inclusion criteria, but which were excluded, and explain why they were excluded. | Figure 2 |
| Study characteristics | 17 | Cite each included study and present its characteristics. | 5,  Table 1 |
| Risk of bias in studies | 18 | Present assessments of risk of bias for each included study. | 5,  eFigure 1 |
| Results of individual studies | 19 | For all outcomes, present, for each study: (a) summary statistics for each group (where appropriate) and (b) an effect estimate and its precision (e.g. confidence/credible interval), ideally using structured tables or plots. | 10-11, Figure 3,  Figure 4,  eFigure 2 |
| Results of syntheses | 20a | For each synthesis, briefly summarise the characteristics and risk of bias among contributing studies. | 5-6, eFigure 1 |
|  | 20b | Present results of all statistical syntheses conducted. If meta-analysis was done, present for each the summary estimate and its precision (e.g. confidence/credible interval) and measures of statistical heterogeneity. If comparing groups, describe the direction of the effect. | 5-6, Figure 3,  Figure 4,  eFigure 2, |
|  | 20c | Present results of all investigations of possible causes of heterogeneity among study results. | 5-6, eFigure 5, eFigure 7 |
|  | 20d | Present results of all sensitivity analyses conducted to assess the robustness of the synthesized results. | 5-6, eFigure 5 |
| Reporting biases | 21 | Present assessments of risk of bias due to missing results (arising from reporting biases) for each synthesis assessed. | 5-6 |
| Certainty of evidence | 22 | Present assessments of certainty (or confidence) in the body of evidence for each outcome assessed. | 5-6,  Table 2 |
| **DISCUSSION** | | |  |
| Discussion | 23a | Provide a general interpretation of the results in the context of other evidence. | 6 |
|  | 23b | Discuss any limitations of the evidence included in the review. | 6-7 |
|  | 23c | Discuss any limitations of the review processes used. | 6-7 |
|  | 23d | Discuss implications of the results for practice, policy, and future research. | 6-7 |
| **OTHER INFORMATION** | | |  |
| Registration and protocol | 24a | Provide registration information for the review, including register name and registration number, or state that the review was not registered. | 2 |
|  | 24b | Indicate where the review protocol can be accessed, or state that a protocol was not prepared. | 2 |
|  | 24c | Describe and explain any amendments to information provided at registration or in the protocol. | 2 |
| Support | 25 | Describe sources of financial or non-financial support for the review, and the role of the funders or sponsors in the review. | 7-8 |
| Competing interests | 26 | Declare any competing interests of review authors. | 7 |
| Availability of data, code and other materials | 27 | Report which of the following are publicly available and where they can be found: template data collection forms; data extracted from included studies; data used for all analyses; analytic code; any other materials used in the review. | 7-8 |

# eTable 2. Search strategy

PubMed

#1

(((((((((((((((((((((((((((((((((((Intracranial hemorrhages[MeSH Terms]) OR (cerebral hemorrhage[MeSH Terms])) OR (Hemorrhages, Intracranial[Title/Abstract])) OR (Intracranial Hemorrhage[Title/Abstract])) OR (Hemorrhage, Intracranial[Title/Abstract])) OR (Posterior Fossa Hemorrhage[Title/Abstract])) OR (Hemorrhage, Posterior Fossa[Title/Abstract])) OR (Hemorrhages, Posterior Fossa[Title/Abstract])) OR (Posterior Fossa Hemorrhages[Title/Abstract])) OR (Brain Hemorrhage[Title/Abstract])) OR (Brain Hemorrhages[Title/Abstract])) OR (Hemorrhage, Brain[Title/Abstract])) OR (Hemorrhages, Brain[Title/Abstract])) OR (Hemorrhage, Cerebrum[Title/Abstract])) OR (Cerebrum Hemorrhage[Title/Abstract])) OR (Cerebrum Hemorrhages[Title/Abstract])) OR (Hemorrhages, Cerebrum[Title/Abstract])) OR (Cerebral Parenchymal Hemorrhage[Title/Abstract])) OR (Cerebral Parenchymal Hemorrhages[Title/Abstract])) OR (Hemorrhage, Cerebral Parenchymal[Title/Abstract])) OR (Hemorrhages, Cerebral Parenchymal[Title/Abstract])) OR (Parenchymal Hemorrhage, Cerebral[Title/Abstract])) OR (Parenchymal Hemorrhages, Cerebral[Title/Abstract])) OR (Intracerebral Hemorrhage[Title/Abstract])) OR (Hemorrhage, Intracerebral[Title/Abstract])) OR (Hemorrhages, Intracerebral[Title/Abstract])) OR (Intracerebral Hemorrhages[Title/Abstract])) OR (Hemorrhage, Cerebral[Title/Abstract])) OR (Cerebral Hemorrhages[Title/Abstract])) OR (Hemorrhages, Cerebral[Title/Abstract])) OR (Brain Hemorrhage, Cerebral[Title/Abstract])) OR (Brain Hemorrhages, Cerebral[Title/Abstract])) OR (Cerebral Brain Hemorrhage[Title/Abstract])) OR (Cerebral Brain Hemorrhages[Title/Abstract])) OR (Hemorrhage, Cerebral Brain[Title/Abstract])) OR (Hemorrhages, Cerebral Brain[Title/Abstract])

#2

((((((((((((magnetic resonance imaging[MeSH Terms]) OR (Tomography[MeSH Terms])) OR (Tomography, Emission-Computed[MeSH Terms])) OR (Tomography, Optical[MeSH Terms])) OR (Tomography, X-rays[MeSH Terms])) OR (magnetic resonance imaging[Title/Abstract])) OR (magnetic resonance[Title/Abstract])) OR (MRI[Title/Abstract])) OR (MR[Title/Abstract])) OR (CT[Title/Abstract])) OR (Radiomics[Title/Abstract])) OR (Radiomic[Title/Abstract])) OR (computed tomography[Title/Abstract])

#3

(((((((((((((((((((((((((((Artificial Intelligence[MeSH Terms]) OR (Deep Learning[MeSH Terms])) OR (Machine Learning[MeSH Terms])) OR (Intelligence, Artificial[Title/Abstract])) OR (Computational Intelligence[Title/Abstract])) OR (Intelligence, Computational[Title/Abstract])) OR (Machine Intelligence[Title/Abstract])) OR (Intelligence, Machine[Title/Abstract])) OR (Computer Reasoning[Title/Abstract])) OR (Reasoning, Computer[Title/Abstract])) OR (AI (Artificial Intelligence[Title/Abstract]))) OR (Computer Vision Systems[Title/Abstract])) OR (Computer Vision System[Title/Abstract])) OR (System, Computer Vision[Title/Abstract])) OR (Systems, Computer Vision[Title/Abstract])) OR (Vision System, Computer[Title/Abstract])) OR (Vision Systems, Computer[Title/Abstract])) OR (Knowledge Acquisition (Computer[Title/Abstract]))) OR (Acquisition, Knowledge (Computer[Title/Abstract]))) OR (Knowledge Representation (Computer[Title/Abstract]))) OR (Knowledge Representations (Computer[Title/Abstract]))) OR (Representation, Knowledge (Computer[Title/Abstract]))) OR (Learning, Machine[Title/Abstract])) OR (Transfer Learning[Title/Abstract])) OR (Learning, Transfer[Title/Abstract])) OR (Learning, Deep[Title/Abstract])) OR (Hierarchical Learning[Title/Abstract])) OR (Learning, Hierarchical[Title/Abstract])

#4

(((((((((((((((hematoma expansion[Title/Abstract]) OR (hemorrhages expansion[Title/Abstract])) OR (hematoma progression[Title/Abstract])) OR (hemorrhages progression[Title/Abstract])) OR (hematoma growth[Title/Abstract])) OR (hemorrhages growth[Title/Abstract])) OR (hematoma enlargement[Title/Abstract])) OR (hemorrhages enlargement[Title/Abstract])) OR (haematoma expansion[Title/Abstract])) OR (haemorrhages expansion[Title/Abstract])) OR (haematoma progression[Title/Abstract])) OR (haemorrhages progression[Title/Abstract])) OR (haematoma growth[Title/Abstract])) OR (haemorrhages growth[Title/Abstract])) OR (haematoma enlargement[Title/Abstract])) OR (haemorrhages enlargement[Title/Abstract])

#1 and (#2 or #3) and #4

# eTable 3. The formula of the outcome measures.

Se =TP/ (TP+ FN)

Sp =FP / (FP + TN)

PPV =TP / (TP + FP)

NPV =TN / (FN + TN)

DAR =(TP+TN)/(TP+FP+FN+TN)

DOR =（TP*TN）/(FP*FN)

TP: true positives; FP: false positives; TN: true negatives; FN: false negatives; Se: sensitivity; Sp: specificity; PPV: positive predictive value; NPV: negative predictive value; DAR: diagnostic accuracy rate; DOR: diagnostic odds ratio.

# eTable 4. The ranking result and the value of surface under the cumulative ranking curve (SUCRA).

| Rank | 1 | 2 | 3 | 4 | 5 | 6 | 7 | 8 | 9 | 10 | 11 | 12 |
| --- | --- | --- | --- | --- | --- | --- | --- | --- | --- | --- | --- | --- |
| Se | Radiomics radiological model | Radiomics model | Heterogeneity | Irregular shape | Radiological model | Hypodensity | Midline shift | Satellite sign | Blend sign | Black hole sign | Island sign | Swirl sign |
|  | 0.914 | 0.899 | 0.7 | 0.699 | 0.69 | 0.578 | 0.501 | 0.329 | 0.27 | 0.207 | 0.109 | 0.102 |
| Sp | Radiomics radiological model | Radiomics model | Irregular shape | Heterogeneity | Radiological model | Hypodensity | Midline shift | Satellite sign | Blend sign | Black hole sign | Island sign | Swirl sign |
|  | 0.914 | 0.898 | 0.701 | 0.7 | 0.688 | 0.579 | 0.504 | 0.324 | 0.271 | 0.207 | 0.11 | 0.103 |
| PPV | Radiomics model | Radiomics radiological model | Island sign | Black hole sign | Radiological model | Swirl sign | Blend sign | Satellite sign | Hypodensity | Midline shift | Heterogeneity | Irregular shape |
|  | 0.865 | 0.846 | 0.645 | 0.628 | 0.59 | 0.574 | 0.565 | 0.454 | 0.312 | 0.272 | 0.161 | 0.087 |
| NPV | Radiomics model | Radiomics radiological model | Radiological model | Hypodensity | Heterogeneity | Irregular shape | Satellite sign | Black hole sign | Blend sign | Island sign | Midline shift | Swirl sign |
|  | 0.947 | 0.944 | 0.721 | 0.569 | 0.535 | 0.423 | 0.411 | 0.364 | 0.331 | 0.319 | 0.258 | 0.176 |
| DAR | Radiomics radiological model | Radiomics model | Radiological model | Black hole sign | Island sign | Blend sign | Hypodensity | Satellite sign | Heterogeneity | Swirl sign | Irregular shape | Midline shift |
|  | 0.923 | 0.767 | 0.687 | 0.659 | 0.619 | 0.499 | 0.442 | 0.398 | 0.395 | 0.325 | 0.201 | 0.085 |

Rank: 1-12 represents the size of the surface under the cumulative ranking curve (SUCRA). From Rank 1 to Rank 12, the value of SUCRA decreases, the worse of diagnostic test accuracy index (Se, SP. PPV, NPV, DAR).

Se: sensitivity; Sp: specificity; PPV: positive predictive value; NPV: negative predictive value; DAR: diagnosis accuracy rate.

# eTable 5. Comparisons of the fit of consistency and inconsistency models.

| Indicator | Consistency model | | | Inconsistency model | | |
| --- | --- | --- | --- | --- | --- | --- |
|  | Dbar | pD | DIC | Dbar | pD | DIC |
| Se | 39.35045 | 30.25927 | 69.60971 | 39.42736 | 30.24156 | 69.66892 |
|  | 37data points, | ratio 1.064, | I^2=0.09 | 37data points, | ratio 1.066, | I^2=0.09 |
| Sp | 39.35975 | 30.27694 | 69.63669 | 35.87886 | 32.35331 | 68.23217 |
|  | 37data points, | ratio 1.064, | I^2=0.09 | 37data points, | ratio 0.9697, | I^2=0 |
| PPV | 39.27806 | 24.09911 | 63.37717 | 38.80856 | 24.30298 | 63.11155 |
|  | 37data points, | ratio 1.062, | I^2=0.08 | 37data points, | ratio 1.049, | I^2=0.07 |
| NPV | 33.36987 | 18.79965 | 52.16952 | 33.9374 | 19.06443 | 53.00183 |
|  | 37data points, | ratio 0.9019, | I^2=0 | 37data points, | ratio 0.9172, | I^2=0 |
| DAR | 38.05782 | 31.64424 | 69.70206 | 37.90968 | 31.59902 | 69.50871 |
|  | 37data points, | ratio 1.029, | I^2=0.05 | 37data points, | ratio 1.025, | I^2=0.05 |

DIC: deviance information criterion

Se: sensitivity; Sp: specificity; PPV: positive predictive value; NPV: negative predictive value; DAR: diagnosis accuracy rate.

# eFigure 1. Risk of bias assessment for included studies (QUADAS-2 tool).


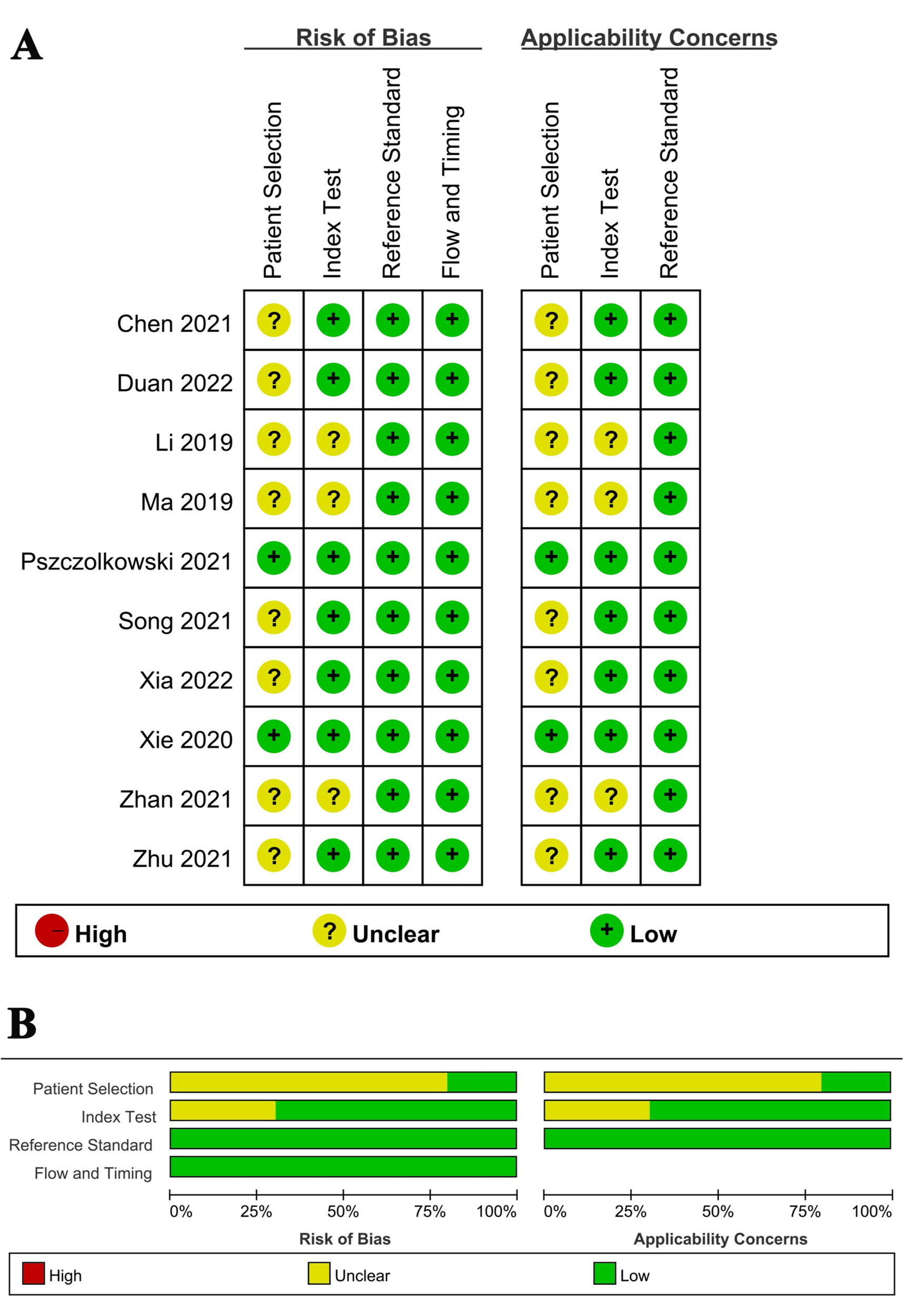


# eFigure 2. The forest plot of diagnostic odd ratio for diagnostic test meta-analysis.


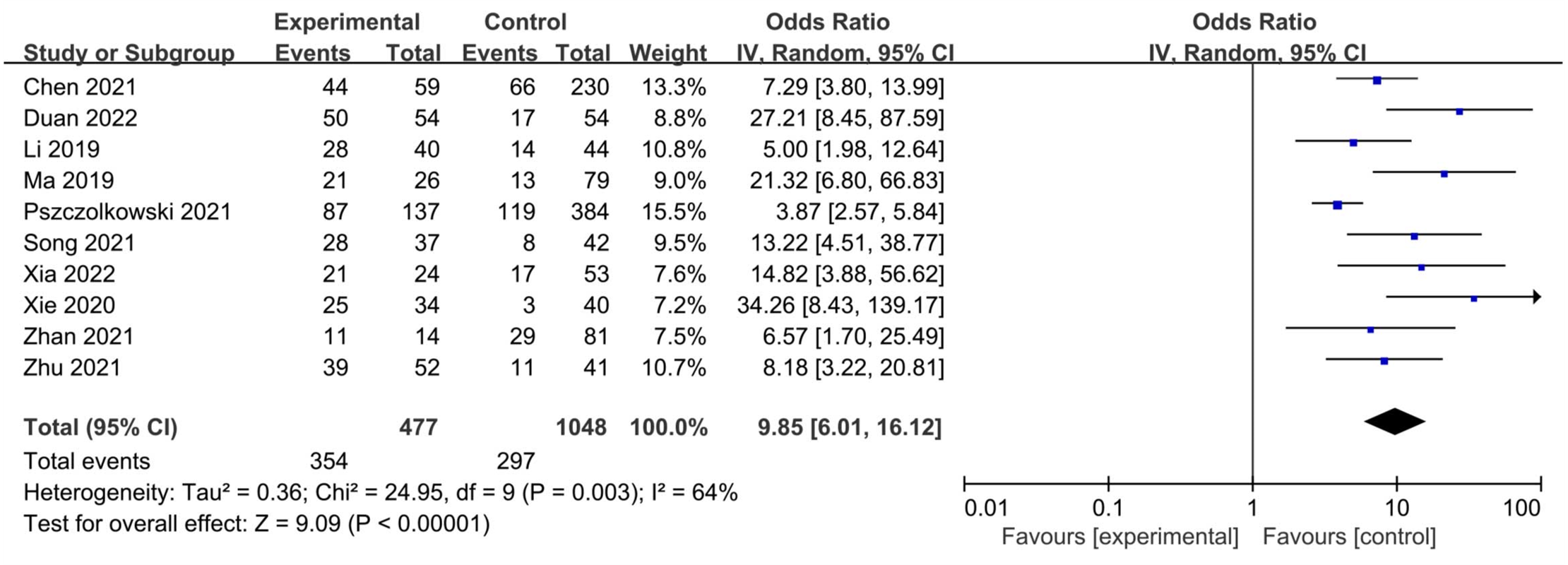


# eFigure 3. Summary receiver operating characteristic curves (sROC) of the screening indicators.


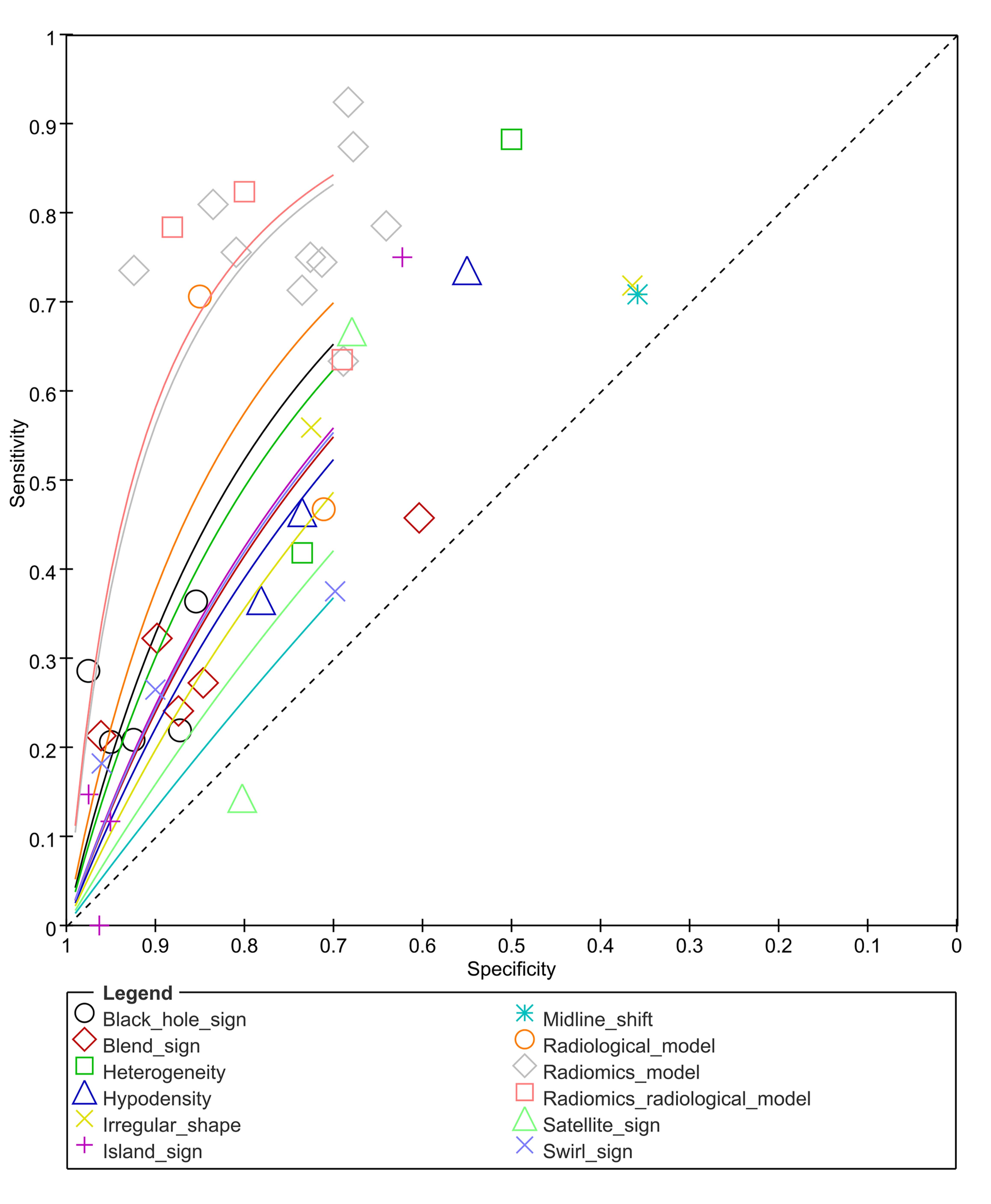


# eFigure 4. Summary receiver operating characteristic curves (sROC) fo radiomics model.


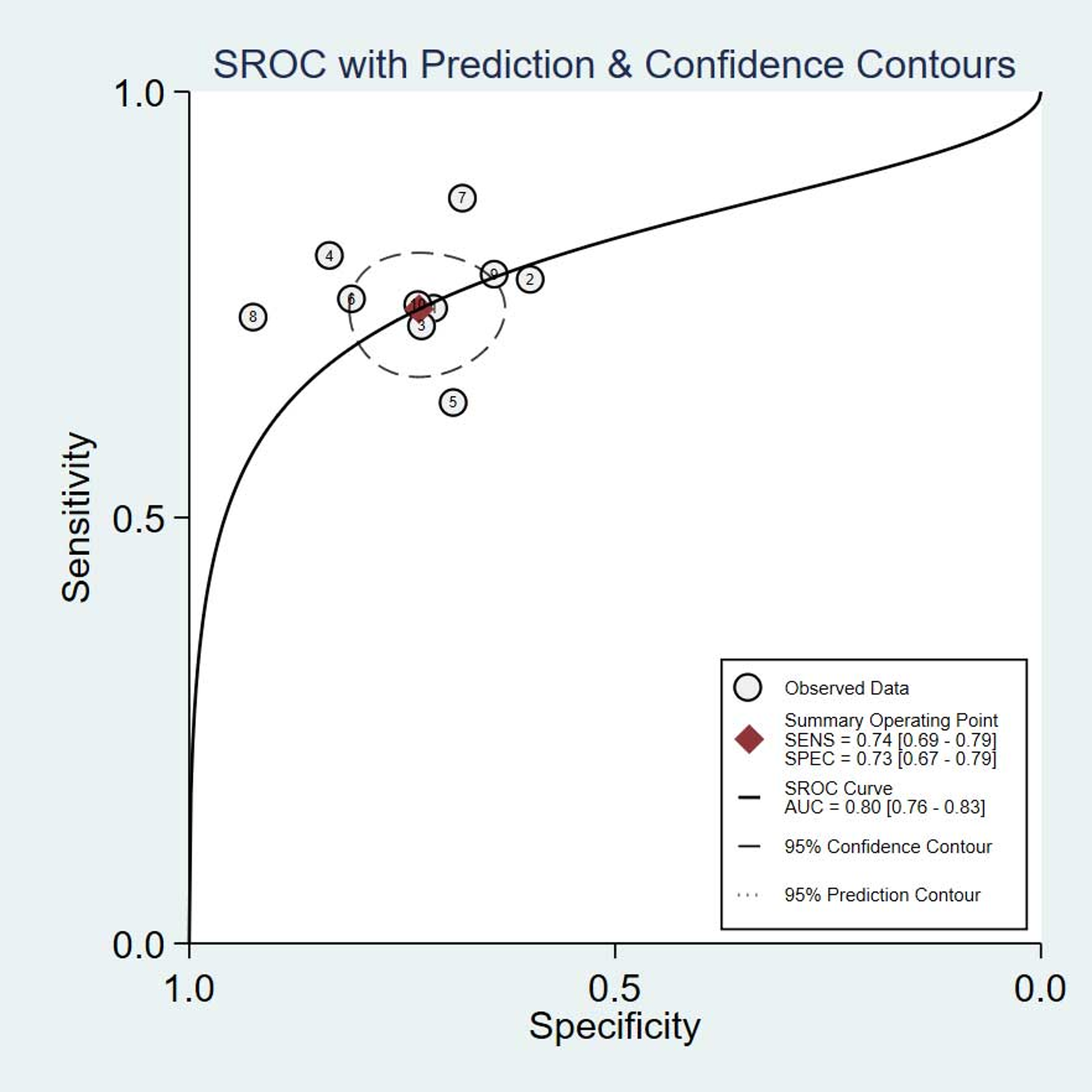


# eFigure 5. Sensitivity analysis for radiomics by omitting each included article one by one.

**A. For sensitivity (Se).**

**
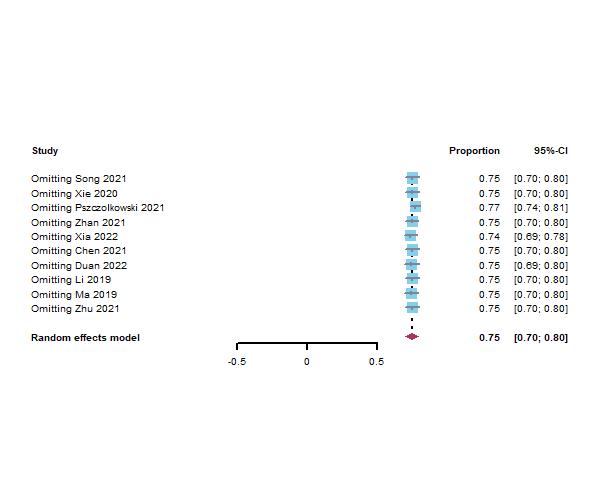
**

**B. For specificity (Sp).**

**
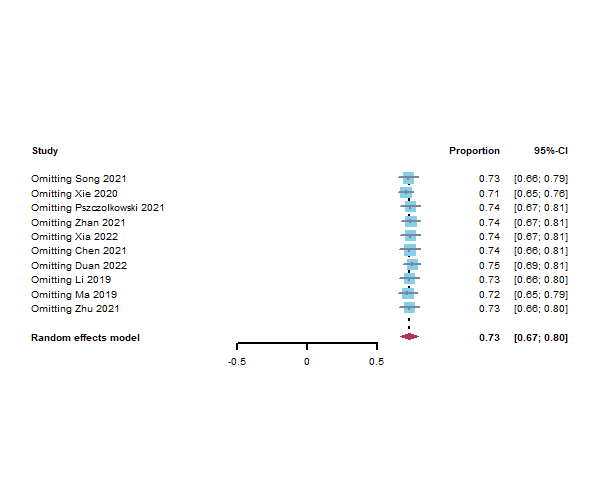
**

**C. For positive prediction value (PPV).**

**
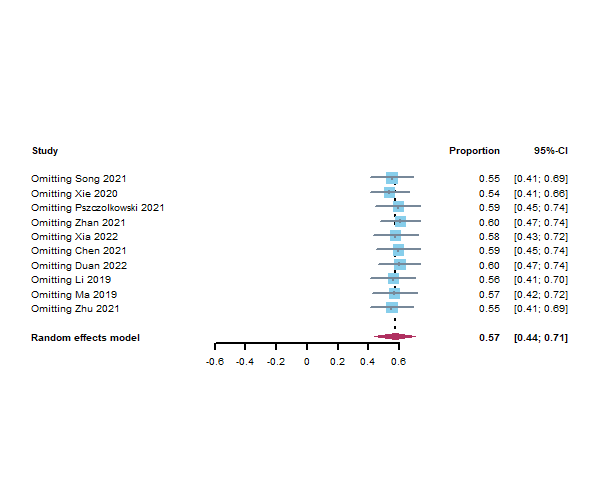
**

**D. For negative prediction value (NPV).**

**
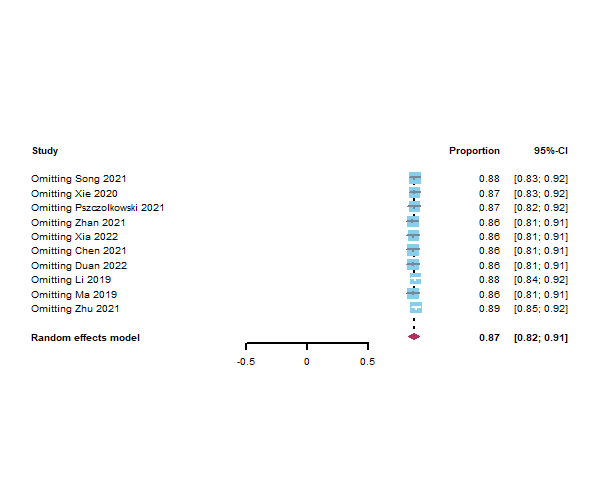
**

**E. For diagnosis accuracy rate (DAR).**

**
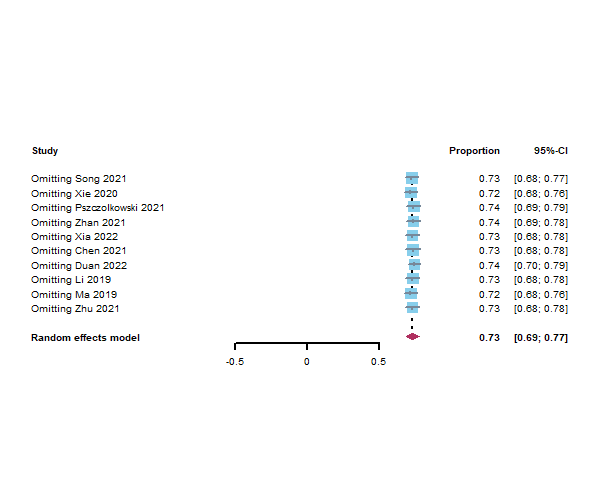
**

# eFigure 6. Funnel plot of publishing bias for radiomics model.

**A. For sensitivity (Se).**

**
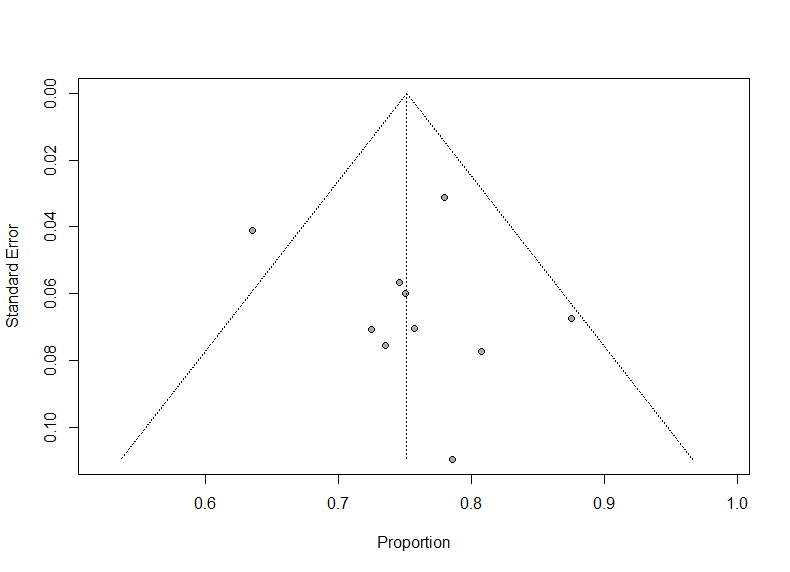
**

**B. For specificity (Sp).**

**
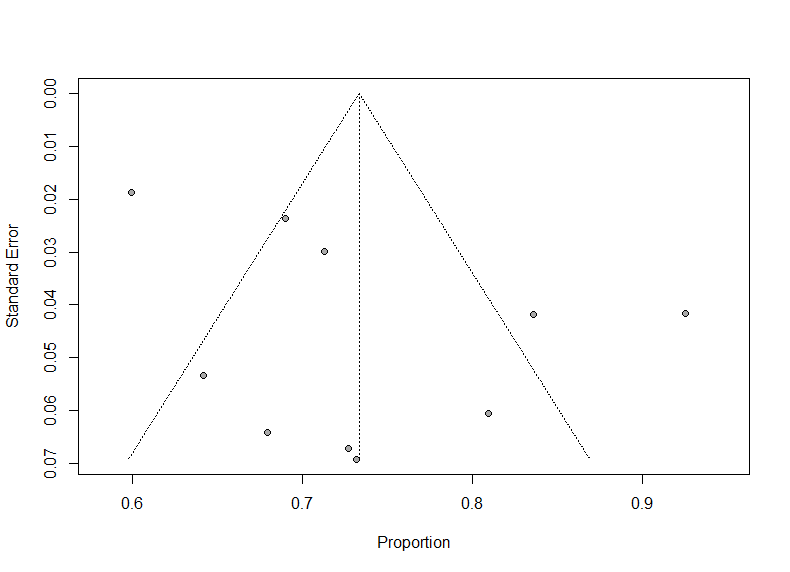
**

**C. For positive predictive value (PPV).**

**
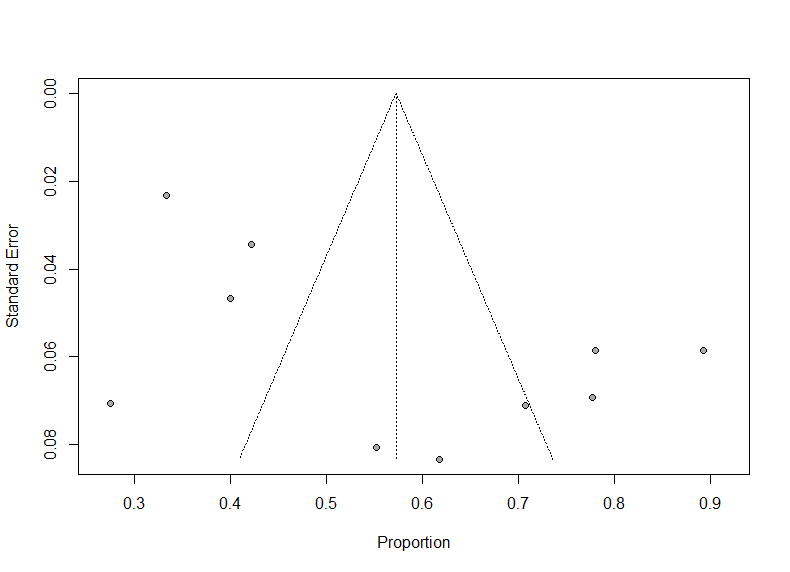
**

**D. For negative predictive value (NPV).**

**
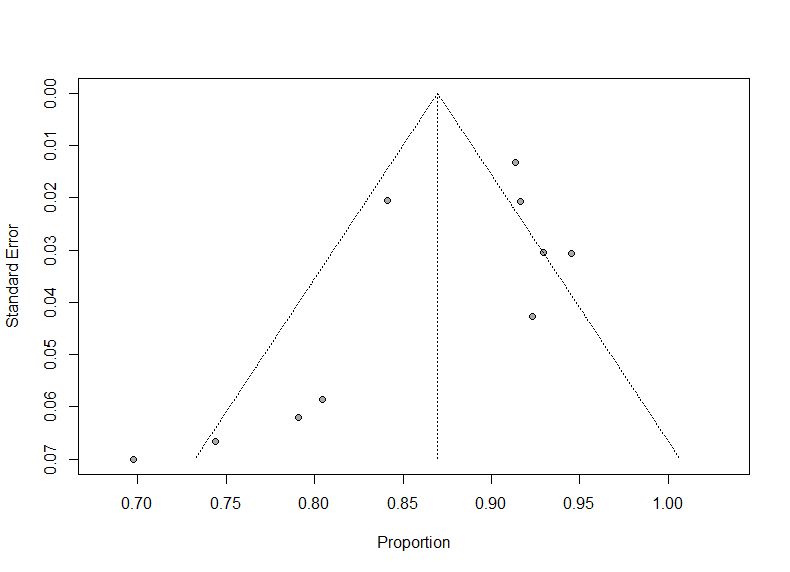
**

**E. For diagnostic accuracy rate (DAR).**

**
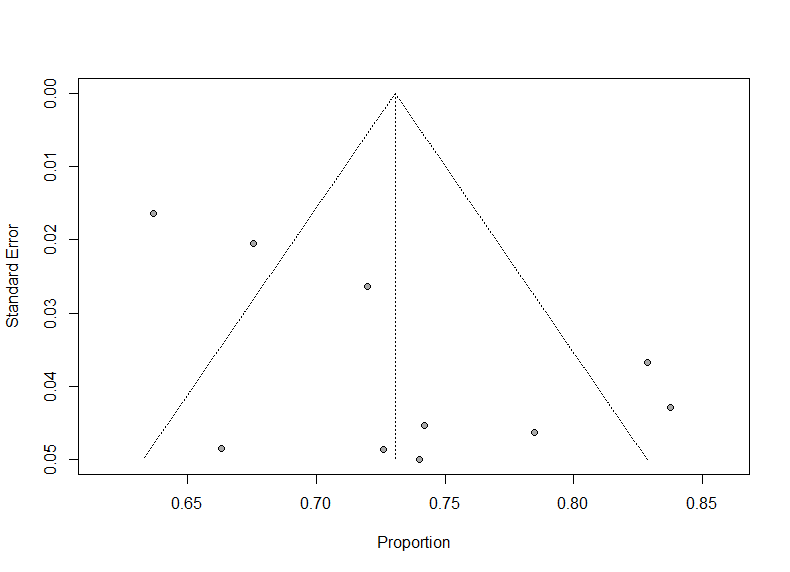
**

**F. For diagnostic odd ratio (DAR).**

**
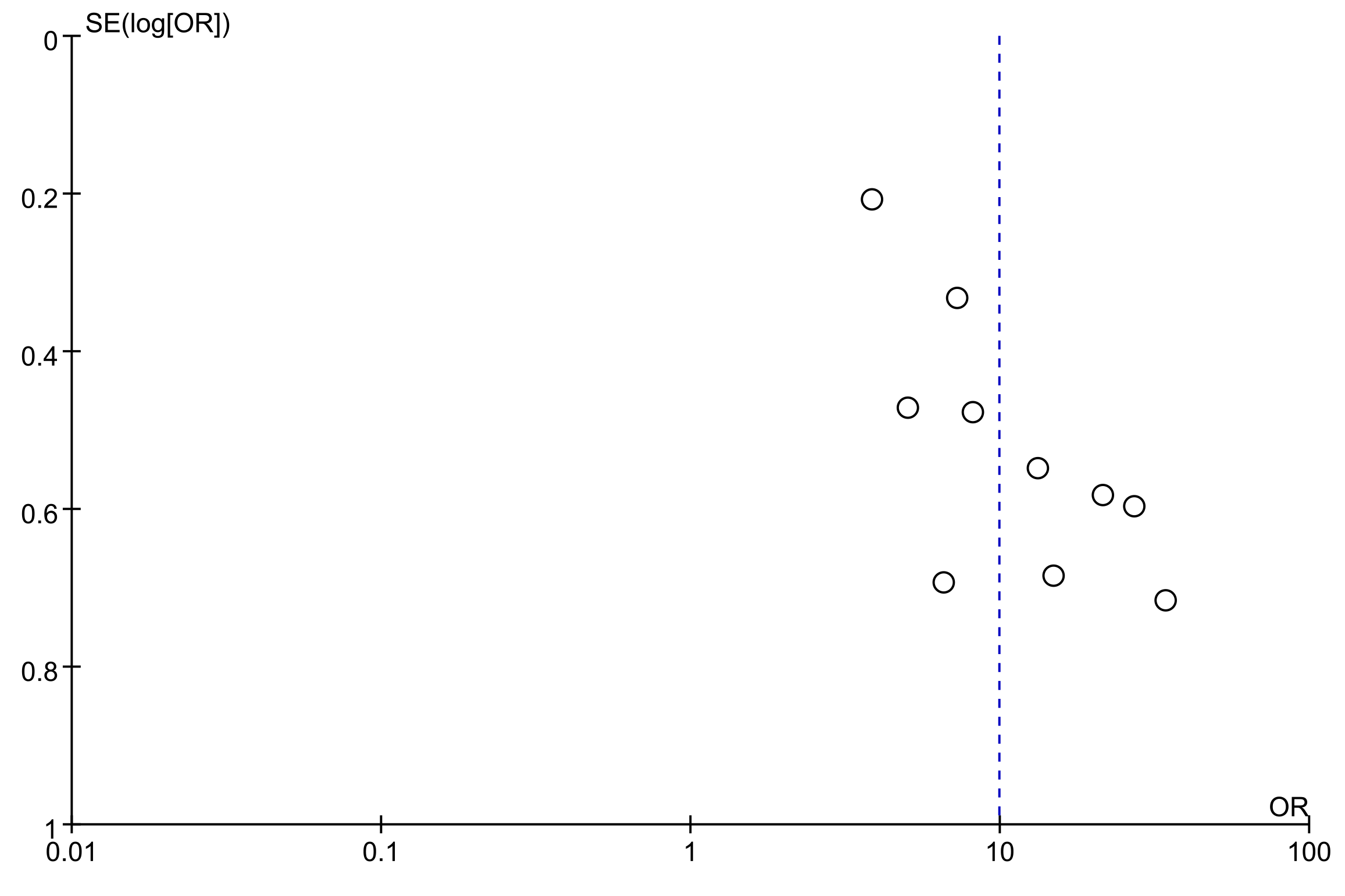
**

# eFigure 7. Node-split for assessing local heterogeneity.

**A. For sensitivity (Se)**

**
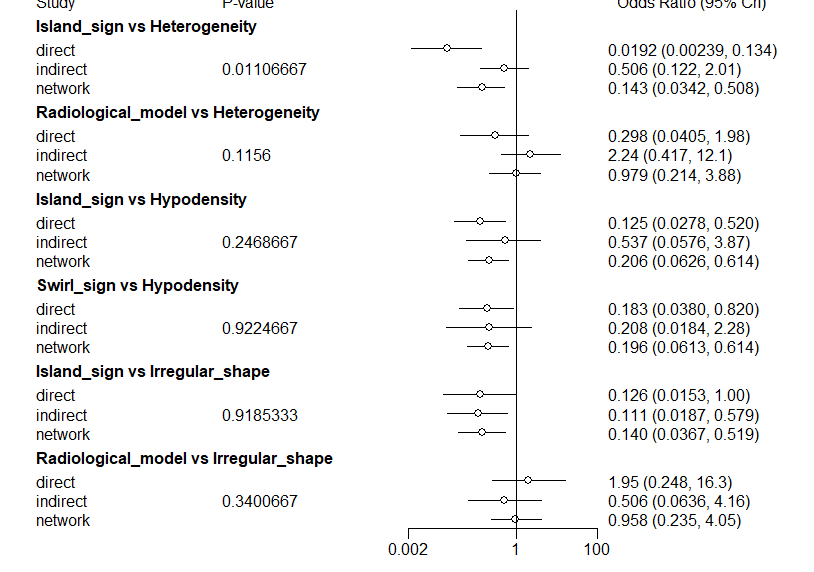

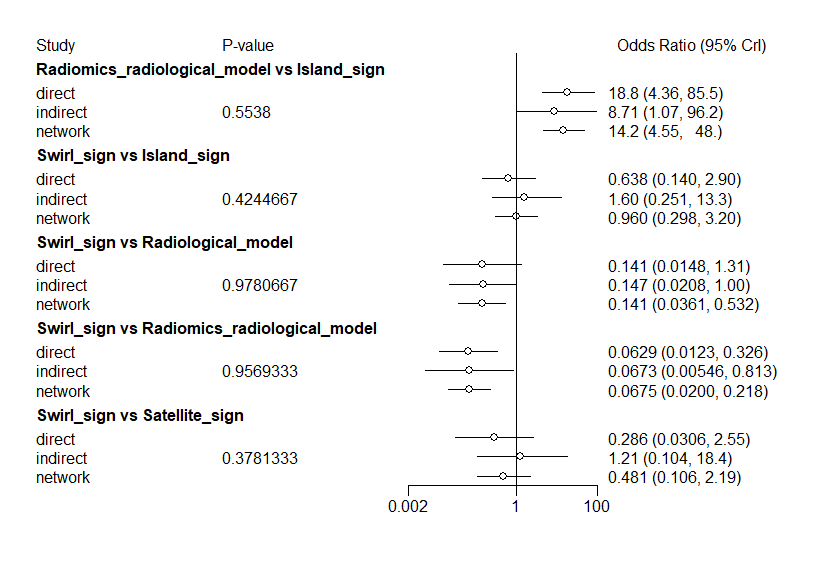
**

**B. For specificity (Sp).**

**
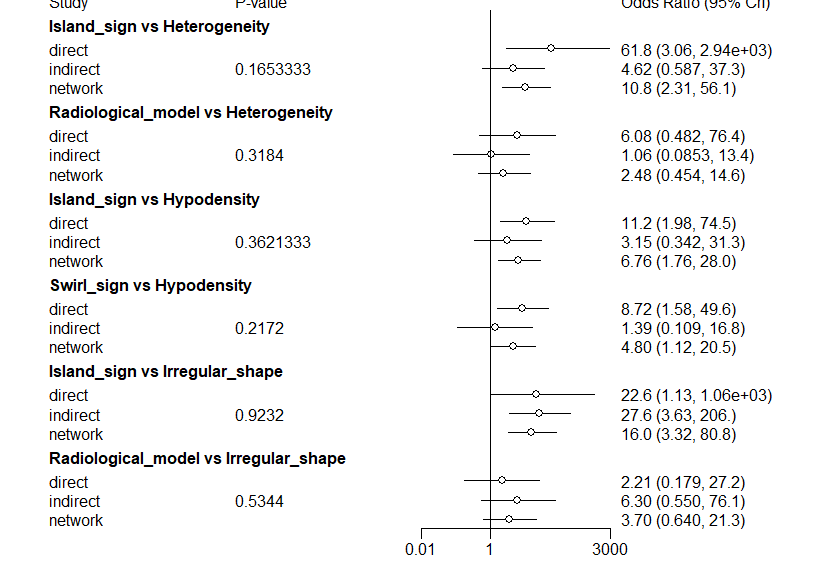

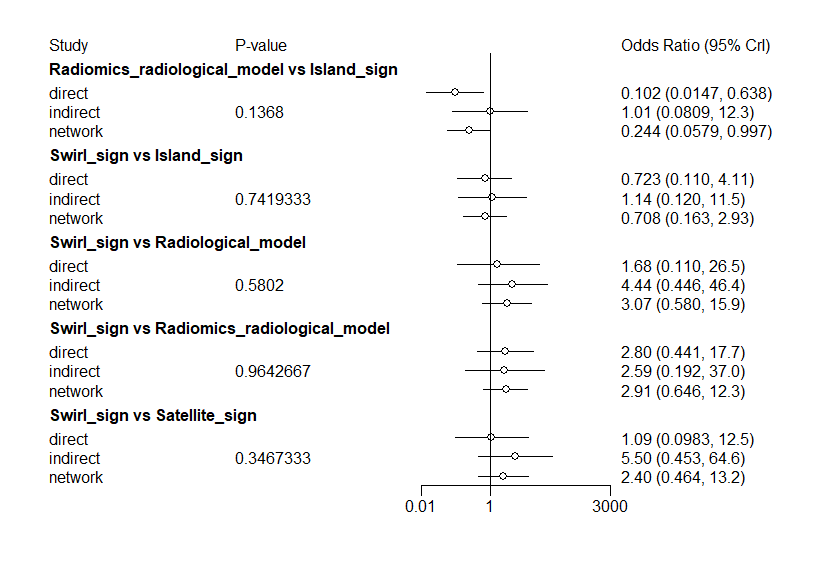
**

**C. For positive predictive value(PPV).**

**
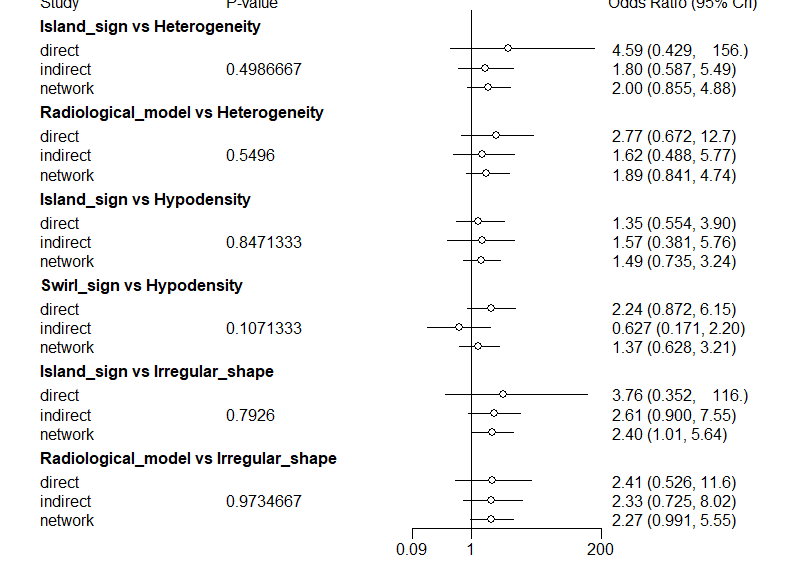
**

**
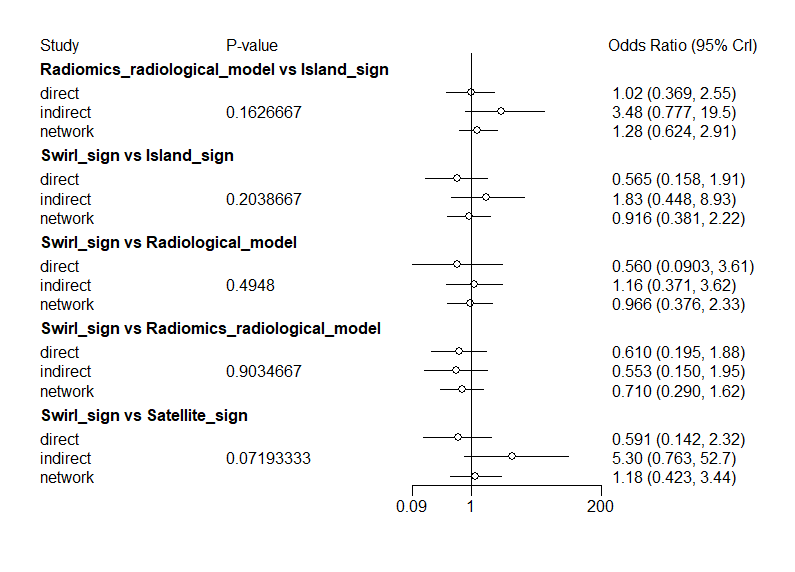
**

**D. For negative predictive value (NPV).**

**
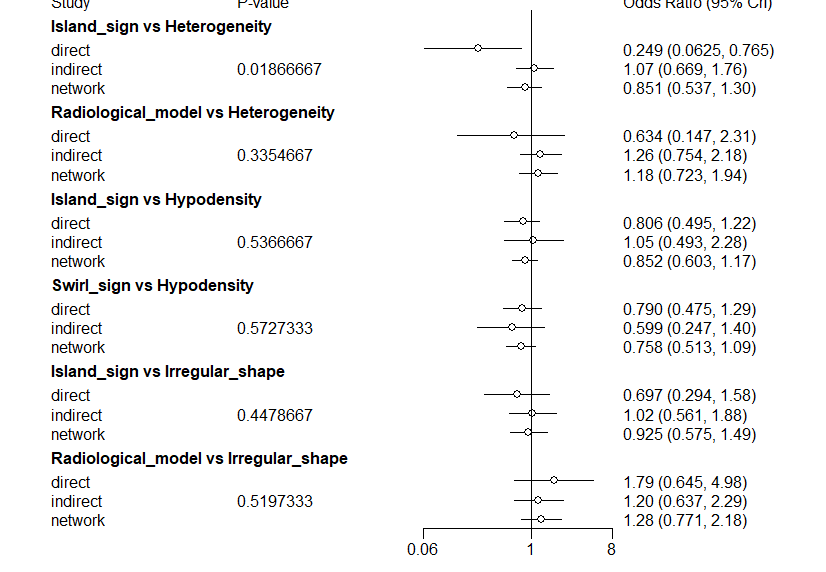
**

**
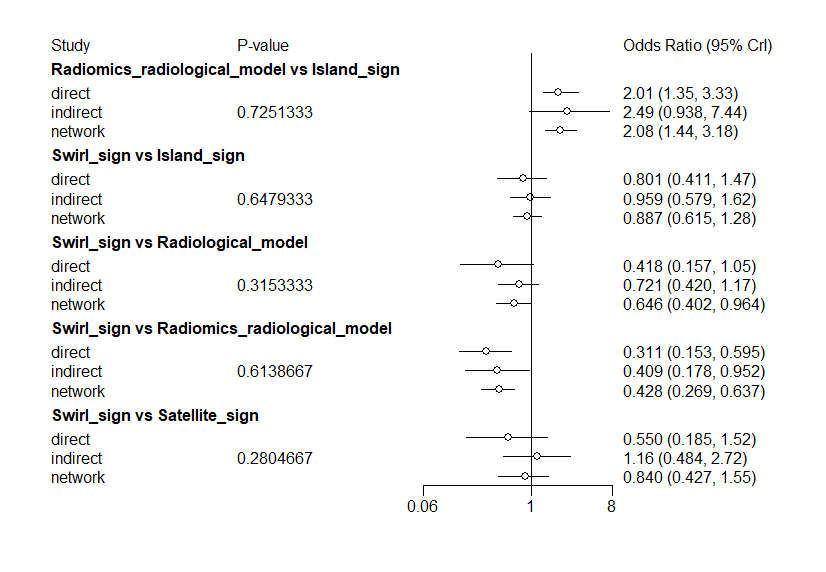
**

**E. For diagnosis accuracy rate (DAR).**

**
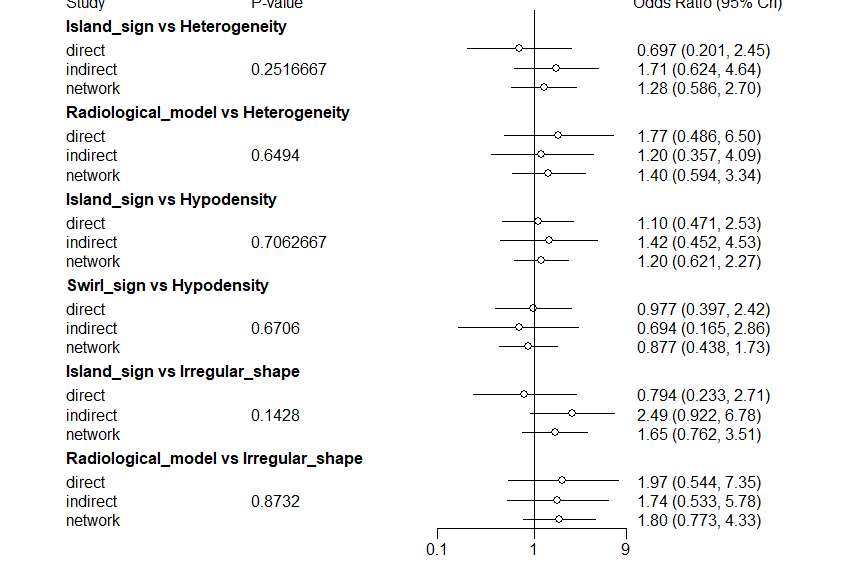
**

**
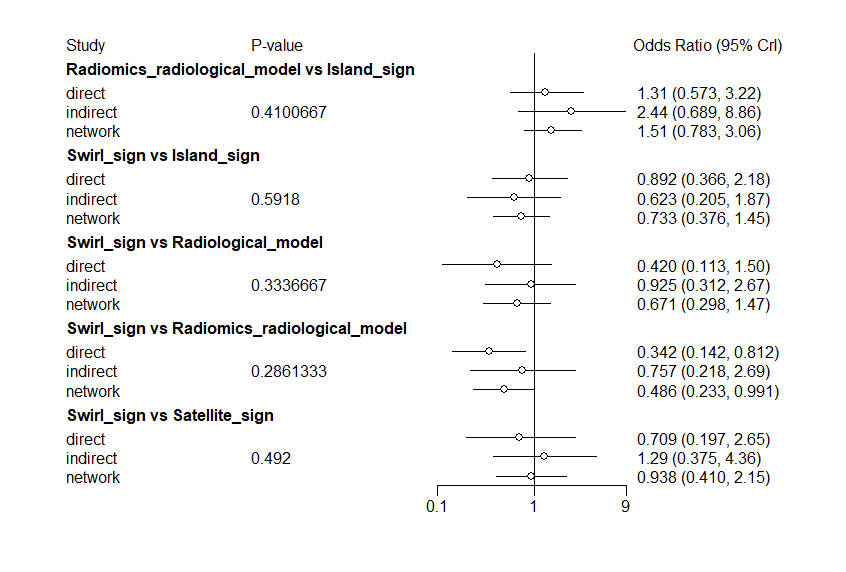
**
